# How to Secure Good Dental Organs, Etc.

**Published:** 1884-07

**Authors:** H. E. Dennett

**Affiliations:** Boston, Mass.


ARTICLE VI.

### HOW TO SECURE GOOD DENTAL ORGANS, PREVENT THEIR DECAY, PREVENT RICKETS, HIP DISEASES, ETC.

BY H. E. DENNETT, D. D. S., BOSTON, MASS.

In the discharge of their duties, the physician and dentist are daily asked by their patients, "What must I do to prevent my teeth from decaying!" The answer should be, "Correct your diet." That is, eat such food, and only such as contains all of its natural elements. If we eat the products of grain, we must eat them with all their elements as furnished by Nature. If we eat meat we must also eat bones or our systems will suffer from a violation of one of Nature's unerring laws. It is conceded that dental decay is the dissolving away of the lime salts by the vitiated secretions. This is not due so much to a want of cleanliness of the mouth as is commonly supposed; for it is not true that "a clean tooth never decays!" One may devote twelve hours out of the twenty-four to the ablution of the mouth, and fail to prevent decay of the teeth so long as Nature's dietetic laws are violated. Dental development in man is discernable as early as the seventh week of intra-uterine life; hence the importance of a strictly correct diet from the start, if mothers wish to give birth to children who may have perfectly formed teeth; and perfect health includes a perfect set of teeth, for they are little indicators, denoting by *their* condition that of the whole system, just as a thermometer indicates thermal changes. A mother who passes through the period of gestation and lactation without a sufficient supply of tooth and bone material in her food will suffer from loss of teeth, neuralgia, rheumatism, and other diseases that result from an impoverished state of the system. The lime from her teeth will be dissolved, taken into the circulation, and appropriated by the offspring. Excepting *civilized* man, all flesh eating animals eat as much of the bone with the flesh they devour as they can break with their teeth sufficiently fine to swallow. Place before a tribe of Indians everything the earth produces in the shape food, and they will eat only animal food so long as that lasts; but put them upon a resservation, and feed them as civilized people feed themselves, and decay of the teeth soon follows. Take from any carnivorous animal its supply of bone which Nature furnishes with the flesh, and decay of the teeth is sure to follow. Several years ago, the lions in the Zoological Gardens of London were fed upon the thighs of horses. These being large and hard, they were unable to break and eat, and as a consequence their young were born with ceft palates and died shortly after birth. They were afterwards fed upon deer and other small animals, and their young were born with perfectly formed palates and lived. Vetinary surgeons have long known that certain diseases of their dumb patients can only be successfully treated by feeding them with bone meal. A dam too aristocratic to gnaw bones, gave birth to successive litters of rickety colts. After being fed with food containing bone meal she produced perfectly healthy ones by the same sire.

Even our domestic herbivorous animals thrive better when bone is added to their bill of fare. The cow who every year gives birth to young has an excessive drain upon her supply of bone material, and craves bone to such an extent that she will try to masticate even very large ones, as every farmer's boy can testify.

Arguments in favor of eating bone to prevent decay of the teeth as well as to cure a long catalogue of bone and kindred diseases might be continued indefinitely, but, as a "word to the wise is sufficient," it seems only necessary to add that a long and continued experiment has been made upon a family, with results that fully justify all claims made for it. The bones were selected from perfectly healthy animals, none being used that bore the slightest blemish, carefully and without being allowed to pass through any perceptible chemical changes, finely granulated, and incorporated into soup, gravey, bread, etc., in the proportion of from one to three spoonsful to each pint of soup, gravey or flour.

The relative proportion of nutritive elements in one hundred parts of different kinds of animal food has been found as follows: Beef, 26; Mutton, 26; Chicken, 27; Pork, 24; Blood, 20; Brain, 21; Codfish, 21; White of egg, 14; Milk, 7; Bone, 51.—*Medical Tribune.*

